# Supplementary material for: In-Silico Analysis of Monoclonal Antibodies against SARS-CoV-2 Omicron
Source: Viruses. 2022 Feb 14;14(2):390. doi: 10.3390/v14020390 (PMC8876352; doi:10.3390/v14020390)
Supplement: Supplementary file 1 [file viruses-14-00390-s001.zip › viruses-1537958-supplementary.pdf]

Table S1. The list of epitopes and paired antibodies.

Group	Antibody	Reference DOI	PDB	Epitope	Source	Reference
NTD	Fab2-17	10.1016/j.chom.2021.03.005	7LQW	Q14,N17,L18,T76,K77,V143	IEDB	[1]
	COVOX-159	10.1016/j.cell.2021.02.032	7NDD	Q14,Y144,Y145,H146,K147,F157,G252,D253	IEDB	[2]
	Fab4-8	10.1016/j.chom.2021.03.005	7LQV	Q14, Y144, H146, K147, N148, N149, W152, M153, E154, E156, F157, R158, R246, Y248, L249, P251, G252, D253	IEDB	[1]
	Fab5-24	10.1016/j.chom.2021.03.005	7L2F	Q14,C15,Y144,H146,K147,E154,E156,R158,R246,Y248,L249,T250,P251,D253,S254	IEDB	[1]
	DH1050.1	10.1016/j.cell.2021.06.021	7LCN	F140,G142,V143,Y145,H146,N148,N149,W152,E154,F157,A243,L244,H245	IEDB	[3]
	S2X333	10.1016/j.cell.2021.03.028	7LXY	V16,N17,F140,G142,V143,Y144,Y145,H146,K147,N148,W152,E154,E156,R158,L244,H245,R246,L249,P251	IEDB	[4]
	S2M28	10.1016/j.cell.2021.03.028	7LY3	V16,N17,T20,Y144,Y145,H146,K147,N148,S155,R158,R246,L249,T250,P251,G252,D253	IEDB	[4]
	S2L28	10.1016/j.cell.2021.03.028	7LXZ	V16,N17,T19,Y144,R246,S247,Y248,T250,P251,G252,D253,S254,S255,S256,G257	IEDB	[4]
	Fab4-18	10.1016/j.chom.2021.03.005	7L2E	Q14,C15,V16,N17,T19,G142,V143,Y144,K147,E156,R158,L244,H245,R246,S247,Y248,L249,T250,P251,G252,S256	IEDB	[1]
	CM25	10.1126/science.abg5268	7M8J	Y144,Y145,H146,K147,R246,S247,Y248,L249,T250,P251,G252,S255	IEDB	[5]
	Fab2-51	10.1016/j.chom.2021.03.005	7L2C	Y144,Y145,H146,K147,N148,K150,R246,S247,Y248,L249,T250,P251,G252,D253,S254	IEDB	[1]
	Fab1-87	10.1016/j.chom.2021.03.005	7L2D	Y144,Y145,H146,K147,K150,W152,H245,R246,Y248,L249,T250,P251,G252,S254, S255,S256	IEDB	[1]
	4A8	10.1126/science.abc6952	7C2L	Y144,Y145,H146,K147,K150,W152,H245,R246,S247,Y248,L249	IEDB	[6]
	FC05	10.1038/s41422-020-00446-w	7CWU	Y144,Y145,H146,K147,K150,W152,R246,S247,Y248,L249,S256	IEDB	[7]
	CM17	10.1126/science.abg5268	N/A	Y145,K147,W152,Y248	Reference Figure	[5]
	CM30	10.1126/science.abg5268	N/A	Y144,W152,R246,Y248	Reference Figure	[5]
	P008_056	10.1126/sciadv.abg7607	7NTC	S71,K97,S98,T124,Y145,H146,K147,K150,S151,W152,E180,G181,K182,Q183,N185,V213,H245,S247,Y248,L249,T259,A260,A262	IEDB	[8]
	DH1052	10.1016/j.cell.2021.06.021	7LAB	A27,Y28,T29,N30,F32,N61,W64,H66,I68,H69,K97,F186,N211,L212,V213,R214,D215,L216,P217,Q218,S605,N606	IEDB	[3]
	CoV2-2490	10.1016/j.cell.2021.05.032	7DZY	N30,F32,W64,H66,I68,K97,N185,K187,N211,V213,R214	IEDB	[9]
RBD-1	CT-P59	10.1038/s41467-020-20602-5	7CM4	R403,K417,Y449,N450,L452,Y453,L455,F456,E484,G485,F486,Y489,F490,L492,Q493,S494,Y495,Y505	IEDB	[10]
	P5A-2G7	10.1038/s41422-021-00487-9	7D03	R403,K417,Y449,L452,Y453,L455,F456,E484,G485,F486,C488,Y489,F490,L492,Q493,Y505	IEDB	[11]
	COVOX-88	10.1016/j.cell.2021.02.032	7ND4	R403,K417,Y453,L455,F456,E484,G485,F486,N487,C488,Y489,Q493,N501,G502, Y505	IEDB	[2]
	S2H14	10.1016/j.cell.2020.09.037	7JX3	R403,V445,G446,Y449,Y453,L455,F456,N487,Y489,Q493,Y495,G496,Q498,P499, T500,N501,G502,Y505	IEDB	[12]
	C121	10.1038/s41586-020-2852-1	7K8X,7K8Y	G404,D405,E406,V407,R408,Q414,T415,Y449,L452,L455,A475,E484,G485,F486,Y489,F490,L492,Q493,S494,V503,G504,Y505,Y508	IEDB	[13]
	C105	10.1016/j.cell.2020.06.025; 10.1038/s41586-020-2456-9	6XCM, 6XCN	D405,R408,T415,G416,Y421,F456,R457,K458,N460,Y473,Q474,A475,G476,F486, N487,T500,N501,G502,Y505	IEDB	[14,15]
	CC12.1	10.1126/science.abd2321	6XC2,6XC3	D405,E406,T415,K417,D420,Y453,L455,F456,R457,A475,S477,F486,Y489,N487,Q493,G496,Q498,T500,N501,G502,Y505	IEDB	[16]
	Ly-CoV016	10.1016/j.xcrm.2021.100255	N/A	D405,K417,D420,L455,F456,N460,I472,Y473,A475,G476,F486,N487,Y489,G504	Reference Figure	[17]
	CV30'	10.1038/s41467-020-19231-9	6XE1	T415,G416,K417,D420,Y421,Y453,L455,F456,R457,K458,S459,N460,Y473,Q474,A475,G476,S477,F486,N487,Y489,Q493,S494,Y495,G496,Q498,T500,N501,G502,Y505	IEDB	[18]
	P5A-3A1	10.1038/s41422-021-00487-9	7D0C	T415,G416,K417,D420,Y421,L455,F456,R457,K458,N460,Y473,Q474,A475,G476,S477,F486,N487,G496,Y505	IEDB	[11]
	SET90-C11	10.1016/j.celrep.2021.109433	7B3O	T415,G416,K417,D420,Y432,Y453,L455,R457,K458,N460,Y473,A475,G476,F486,N487,Y489,S494,Y495,G496,Q498,T500,N501,G502,Y504	IEDB	[19]
	BG1-22	10.1016/j.cell.2021.04.032	7M6F	T415,Y421,A475,G476,N487,S494,G502	IEDB	[20]
	C105'	10.1038/s41467-021-24435-8	N/A	K417,D420,L455,F456,N460,Y473,A475	Reference Figure	[21]
	REGN10933	10.1126/science.abd0827	6XDG	K417,Y453,L455,F456,E484,G485,F486,N487,C488,Y489,Q493	IEDB	[22]
	P5A-2F11	10.1038/s41422-021-00487-9	7CZZ	Y421,F456,R457,Y473,A475,G476,S477,E484,G485,F486,N487,Y489,Q493	IEDB	[11]
	BG7-15	10.1016/j.cell.2021.04.032	7M6G	N439,N440,S443,K444,V445,G446,G447,Y449,N450,S494,P499,T500,Q506	IEDB	[20]
	COVOX-75	10.1016/j.cell.2021.02.032	7BEO	N440,L441,S443,K444,V445,G446,G447,N448,Y449,N450,L452,F490,L492,Q493,S494,Y495,G496	IEDB	[2]
	C119	10.1038/s41586-020-2852-1	7K8W	K444,V445,G446,Y449,N450,E484,Q493,S494,Q498,G504, Y505	IEDB	[13]
	BD-368-2	10.1016/j.cell.2020.09.035	7CHH,7CHF,7CHC,7CHE	K444,G446,Y449,N450,L452,N481,G482,V483,E484,G485,F490	IEDB	[23]
	C104	10.1038/s41586-020-2852-1	7K8U	R408,K444,T470,V483,F486,F490,V503,G504	IEDB	[13]
	P2C-1A3	10.1038/s41467-020-20501-9	7CDJ	V445,G446,Y449,F456,T478,N481,V483,E484,G485,F486,N487,Y489,F490,L492,Q493,S494,Q498,T500	IEDB	[24]
	CV05-163	10.1126/science.abh1139	7LOP	G446,G447,Y449,F456,T470,V483,E484,G485,F486,C488,Y489,F490,P491,L492,Q493,S494,Q498	IEDB	[25]
	P5A-1B9	10.1038/s41422-021-00487-9	7CZX	G446,N448,Y449,L452,E484,G485,F486,N487,Y489,F490,L492,Q493,S494	IEDB	[11]
	Fab2-15	10.1016/j.celrep.2021.108950	7L5B	G446,Y449,L452,T478,V483,E484,G485,F486,N487,Y489,F490,L492,Q493,S494,G496,Q498	IEDB	[26]
	S2H13	10.1016/j.cell.2020.09.037	7JV2,7JV4,7JV6	G446,Y449,N481,G482,V483,E484,G485,F486,F490,S494	IEDB	[12]
	BD-23	10.1016/j.cell.2020.05.025	7BYR	G446,Y449,E484,G485,F486,Y489,F490,L492,Q493,S494,G496,Q498,N501,Y505	IEDB	[27]
	CV07-250	10.1016/j.cell.2020.09.049	6XKQ	G446,Y449,Y453,L455,F456,A475,G476,S477,T478,G485,F486,N487,Y489,Q493,Y495,Q498,N501,Y505	IEDB	[28]
	COVA2-39	10.1016/j.celrep.2020.108274	7JMP	G446,Y449,F456,A475,V483,E484,G485,F486,N487,Y489,Q493	IEDB	[29]
	DH1041	10.1016/j.cell.2021.06.021	7LAA	Y449,L452,T470,E471,I472,N481,G482,V483,E484,G485,F486,F490,L492,Q493,S494	IEDB	[3]
	Fab2-4	10.1038/s41586-020-2571-7	6XEY	Y449,Y453,L455,F456,E484,G485,F486,Y489,F490,L492,Q493,S494	IEDB	[30]
	H4	10.1016/j.celrep.2021.108950	7L58	Y449,L455,F456,V483,E484,G485,F486,Y489,F490,Q493,S494	IEDB	[26]
	COVOX-316	10.1016/j.cell.2021.02.032	7ND7	Y449,L455,F456,V483,E484,G485,F486,Y489,F490,L492,Q493,S494	IEDB	[2]
	COVOX-384	10.1016/j.cell.2021.02.032	7ND8	L452,L455,F456,I472,N481,G482,V483,E484,G485,F486,Y489,F490	IEDB	[2]
	Ly-CoV555	10.1016/j.xcrm.2021.100255	N/A	L452,I472,V483,E484,G485,F486,F490,Q493,S494	Table	[17]
	COVOX-253	10.1016/j.cell.2021.02.032	7BEN	L455,F456,K458,Y473,A475,G476,S477,T478,G485,F486,N487,Y489,Q493	IEDB	[2]
	COVOX-253H55L	10.1016/j.cell.2021.02.032	7NDA	L455,K458,Y473,A475,G476,S477,T478,G485,F486,N487,C488,Y489,Q493	IEDB	[2]
	C002'	10.1038/s41467-021-24435-8	N/A	L455,F456,E484,F486,F490,Q493	Reference Figure	[21]
	S2E12	10.1126/science.abe3354	7K45,7K4N	L455,Y473,A475,G476,S477,E484,G485,F486,N487,C488,Y489	IEDB	[31]
	BD-623	10.1038/s41422-021-00514-9	7E7Y	F456,Y473,A475,G476,S477,T478,V483,E484,G485,F486,N487,C488,Y489	IEDB	[32]
	COVOX-253H165L	10.1016/j.cell.2021.02.032	7NDB	Y473,A475,T478,F486,N487	IEDB	[2]
	P5A-3C12	10.1038/s41422-021-00487-9	7D0D	V483,E484,F486,Y489	IEDB	[11]
	AZD8895	10.1038/s41564-021-00972-2	7L7E	L455,F456,A475,G476,S477,T478,P479,E484,G485,C488,F486,Y489,Q493	IEDB	[33]

RBD-2	C002	10.1038/s41586-020-2852-1	7K8S,7K8T	A372,N440,K444,V445,Y449,N450,L455,T470,E471,N481,G482,V483,E484,G485,F486,Y489,F490,Q493,S494,T500	IEDB	[13]
	B38-New	10.1038/s41467-020-19231-9	7BZ5	R403,D405,E406,R408,Q409,T415,G416,K417,D420,Y421,Y449,Y453,L455,F456,R457,K458,S459,N460,Y473,Q474,A475,G476,S477,E484,F486,N487,Y489,F490,L492,Q493,S494,Y495,G496,Q498,T500,N501,G502,V503,G504,Y505	IEDB	[18]
	CB6-New	10.1038/s41467-020-19231-9	7C01	R403,D405,E406,R408,Q409,T415,G416,K417,D420,Y421,Y449,Y453,L455,F456,R457,K458,S459,N460,Y473,Q474,A475,G476,S477,F486,N487,Y489,F490,Q493,S494,Y495,G496,Q498,T500,N501,G502,G504,Y505	IEDB	[18]
	COVOX-158	10.1016/j.cell.2021.02.032	7ND6	R403,D405,E406,R408,Q409,T415,G416,K417,D420,Y421,Y453,L455,F456,R457,K458,N460,Y473,Q474,A475,G476,S477,F486,N487,Y489,Y495,G496,Q498,T500,N501,G502,V503,Y505	IEDB	[2]
	CB6	10.1038/s41586-020-2381-y	7C01	R403,D405,E406,R408,Q409,T415,G416,K417,D420,Y421,L455,F456,R457,K458,N460,Y473,Q474,A475,G476,S477,F486,N487,Y489,Q493,Y495,G502,Y505	IEDB	[34]
	P2B-1A10	10.1038/s41422-021-00487-9	7CZQ	R403,D405,E406,R408,T415,G416,K417,D420,Y421,Y449,L455,F456,R457,K458,N460,Y473,Q474,A475,G476,S477,N487,Y489,Q493,S494,G496,T500,N501,G502,V503,Y505	IEDB	[11]
	P5A-1B6	10.1038/s41422-021-00487-9	7CZV	R403,D405,R408,Q409,T415,G416,K417,Y421,Y449,Y453,L455,F456,G485,F486,N487,Y489,Q493,S494,Y495,G496,N501,Y505	IEDB	[11]
	COVOX-150	10.1016/j.cell.2021.02.032	7ND5	R403,D405,R408,T415,G416,K417,D420,Y421,Y453,L455,R457,K458,N460,Y473,Q474,A475,G476,S477,F486,N487,Y489,Q493,Y495,G496,Q498,T500,N501,G502,Y505	IEDB	[2]
	BD-604-New	10.1038/s41422-021-00514-9	7E8F	R403,D405,T415,G416,K417,D420,Y421,Y453,L455,F456,R457,K458,S459,N460,Y473,A475,G476,S477,F486,N487,Y489,Q493,G496,Q498,T500,N501,G502,Y505	IEDB	[32]
	BD-629	10.1016/j.cell.2020.09.035	7CH5,7CHC	R403,D405,T415,G416,K417,D420,Y421,Y453,L455,F456,R457,K458,N460,Y473,Q474,A475,G476,S477,T478,F486,N487,Y489,Q493,N501,Y505	IEDB	[23]
	Ly-CoV488 (Ab133)	10.1126/scitranslmed.abf1906	7KMH	R403,D405,T415,G416,K417,D420,Y421,L455,F456,R457,K458,S459,N460,Y473,Q474,A475,G476,S477,F486,N487,Y489,N501,G502,Y505	IEDB	[35]
	CC12.3	10.1126/science.abd2321	6XC4,6XC7	R403,D405,T415,G416,K417,D420,Y421,Y453,L455,F456,R457,K458,N460,Y473,A475,G476,S477,F486,N487,Y489,Y495,N501,Y505	IEDB	[16]
	COVA2-04	10.1016/j.celrep.2020.108274	7JMO	R403,D405,T415,G416,K417,D420,Y421,Y453,L455,F456,R457,K458,N460,Y473,A475,G476,S477,F486,N487,Y489,Q493,S494,Y495,G496,T500,N501,G502,Y505	IEDB	[29]
	CC12.3-New	10.1126/science.abe6230	7KN7	R403,T415,G416,K417,D420,Y421,Y453,L455,F456,R457,K458,N460,Y473,Q474,A475,G476,F486,N487,Y489,Y495,G496,N501,Y505	IEDB	[36]
	910-30	10.1016/j.celrep.2021.109771	7KS9	R403,D405,T415,G416,K417,D420,Y421,Y453,L455,F456,R457,K458,N460,Y473,A475,G476,F486,N487,Y489,Q493,S494,Y495,G496,Q498,T500,N501,G502,G504,Y505	IEDB	[37]
	CV30	10.1038/s41467-020-19231-9	6XE1	R403,D405,T415,G416,K417,D420,Y421,Y453,L455,F456,R457,K458,S459,N460,Y473,Q474,A475,G476,S477,F486,N487,Y489,F490,Q493,S494,Y495,G496,Q498,T500,N501,G502,Y505	IEDB	[18]
	BD-508	10.1038/s41422-021-00514-9	7E86	R403,D405,T415,G416,K417,D420,Y421,L455,F456,R457,K458,N460,Y473,A475,G476,S477,E484,F486,N487,Y489,F490,Q493,S494,G496,Q498,N501,G502,Y505	IEDB	[32]
	P2B-1A1	10.1038/s41422-021-00487-9	7CZP	R403,E406,R408,Q409,G416,K417,Y449,Y453,L455,F456,F486,N487,Y489,Q493,S494,Y495,G496,Q498,T500,N501,G502,Y505	IEDB	[11]
	COVOX-269	10.1016/j.cell.2021.02.032	7BEM	R403,E406,Q409,T415,G416,K417,D420,Y421,Y453,L455,F456,R457,K458,N460,Y473,Q474,A475,G476,F486,N487,Y489,Q493,S494,Y495,G496,Q498,T500,N501,G502,Y505	IEDB	[2]
	P5A-2G9	10.1038/s41422-021-00487-9	7CZT	R403,R408,Q409,Q414,T415,G416,K417,D420,Y421,G446,Y449,F456,A475,G476,S477,F486,N487,Y489,Q493,S494,G496,Q498,N501,Y505	IEDB	[11]
	C1A-B3	10.1016/j.cell.2021.03.027	7KFW	R403,R408,T415,G416,K417,D420,Y421,Y453,L455,F456,R457,K458,N460,Y473,Q474,A475,G476,F486,N487,Y489,Q493,S494,Y495,G496,Q498,T500,N501,G502,Y505	IEDB	[38]
	B38	10.1126/science.abc2241	7BZ5	R403,Q409,T415,G416,K417,D420,Y421,L455,F456,R457,K458,S459,N460,Y473,Q474,A475,G476,S477,F486,N487,Y489,F490,Q493,Y495,G496,Q498,N501,G502,Y505	IEDB	[39]
	P2C-1F11	10.1038/s41467-020-20501-9	7CDI	R403,T415,G416,K417,D420,Y421,Y453,L455,F456,R457,K458,S459,N460,Y473,Q474,A475,G476,S477,F486,N487,Y489,Q493,G502,Y505	IEDB	[24]
	COVOX-40	10.1016/j.cell.2021.02.032	7ND3	R403,T415,G416,K417,D420,Y421,Y453,L455,F456,R457,K458,S459,N460,Y473,Q474,A475,G476,S477,F486,N487,Y489,Y495,G496,Q498,T500,N501,G502,Y505	IEDB	[2]
	BG4-25	10.1016/j.cell.2021.04.032	7M6D	R403,T415,G416,K417,D420,Y421,Y453,L455,F456,R457,K458,N460,Y473,Q474,A475,G476,S477,F486,N487,Y489,Q493,Y505	IEDB	[20]
	P4A1	10.1038/s41467-021-22926-2	7CJF	R403,T415,G416,K417,D420,Y421,Y453,L455,F456,R457,K458,N460,Y473,Q474,A475,G476,S477,F486,N487,Y489,Q493,S494,Y495,G496,Q498,T500,N501,G502,Y505	IEDB	[40]
	C1A-C2	10.1016/j.cell.2021.03.027	7KFX	R403,T415,G416,K417,D420,Y421,Y453,L455,F456,R457,K458,N460,Y473,Q474,A475,G476,F486,N487,Y489,Q493,S494,Y495,G496,Q498,T500,N501,G502,Y505	IEDB	[38]
	BD-236	10.1016/j.cell.2020.09.035	7CHB,7CHE	R403,T415,G416,K417,D420,Y421,Y453,L455,F456,R457,K458,N460,Y473,A475,G476,S477,F486,N487,Y489,Q493,S494,Y495,G496,Q498,T500,N501,G502,V503,Y505	IEDB	[23]
	C1A-F10	10.1016/j.cell.2021.03.027	7KFY	R403,T415,G416,K417,D420,Y421,Y453,L455,F456,R457,K458,N460,Y473,A475,G476,S477,F486,N487,Y489,Q493,Q498,T500,N501,G502,Y505	IEDB	[38]
	C102	10.1038/s41586-020-2852-1	7K8M	R403,T415,G416,K417,D420,Y421,Y453,L455,F456,R457,K458,N460,Y473,A475,G476,S477,F486,N487,Y489,T500,N501,G502,Y505	IEDB	[13]
	C1A-B12	10.1016/j.cell.2021.03.027	7KFV	R403,T415,G416,K417,D420,Y421,Y453,L455,F456,R457,K458,N460,Y473,A475,G476,F486,N487,Y489,Q493,S494,Y495,G496,Q498,T500,N501,G502,Y505	IEDB	[38]
	BD-604	10.1016/j.cell.2020.09.035	7CH4,7CHF	R403,T415,G416,K417,D420,Y421,Y453,L455,R457,K458,S459,N460,Y473,Q474,A475,G476,S477,F486,N487,Y489,Q493,Q498,T500,N501,G502,V503,Y505	IEDB	[23]
	Ly-CoV481 (Ab128)	10.1126/scitranslmed.abf1906	7KMI	R403,T415,G416,K417,D420,Y421,Y453,L455,R457,K458,N460,Y473,A475,G476,S477,F486,N487,Y489,Q493,S494,Y495,G496,Q498,T500,N501,G502,V503,Y505	IEDB	[35]
	P5A-1B8	10.1038/s41422-021-00487-9	7D00	R403,T415,G416,K417,D420,Y421,L455,F456,R457,N460,Y473,A475,G476,F486,N487,Y489,Q493,G496,T500,N501,G502,Y505	IEDB	[11]
	BD-515	10.1038/s41422-021-00514-9	7E88	R403,T415,K417,D420,Y421,L455,R457,K458,N460,Y473,Q474,A475,G476,S477,T478,F486,N487,Y489,N501,G502,Y505	IEDB	[32]
RBD-3	S309	10.1038/s41586-020-2349-y	6WPS,6WPT	T333,N334,L335,P337,G339,E340,V341,N343,A344,T345,K346,E354,K356,R357,I358,S359,N360,C361,N440,L441,K444,R509	IEDB	[41]
	S309-New	10.1016/j.cell.2020.09.037	7JX3	N334,L335,P337,G339,E340,N343,A344,T345,R346,K356,R357,S359,C361,L441	IEDB	[12]
	BG10-19	10.1016/j.cell.2021.04.032	7M6E	G339,F342,N343,T345,R346,V367,L368,S371,S373,F374,W436,N437,S438,N440,L441,K444,N448,Y449,N450,Q498	IEDB	[20]
	BG7-20	10.1016/j.cell.2021.04.032	7M6H	S375,T376,K378,R408,Q409,Q414,K417,Y449,L452,L455,F456,A475,G482,E484,G485,F486,Y489,F490,L492,Q493,S494,V503,G504,Y505,Q506,Y508	IEDB	[20]
	C144	10.1038/s41586-020-2852-1	7K90	G339,F342,N343,V367,S371,A372,S373,F374,Y449,L455,F456,V483,E484,G485,F486,N487,Y489,F490,Q493,S494	IEDB	[13]
	S2M11-New	10.1016/j.cell.2021.03.028	7LY2	F342,N343,L368,S371,A372,S373,F374,W436,L441,K444,G446,Y449,L452,L455,F456,E484,G485,F486,Y489,F490,L492,Q493,S494	IEDB	[4]
	S2M11	10.1126/science.abe3354	7K43	F342,N343,L368,S371,A372,S373,W436,N440,L441	IEDB	[31]
	CV38-142	10.1016/j.chom.2021.04.005	7LM8	N343,A344,T345,R346,S373,W436,N437,N440,L441,S443,K444,V445,N448,N450,R509	IEDB	[42]
	AZD1061	10.1038/s41564-021-00972-2	7L7D	T345,R346,N439,N440,L441,S443,K444,V445,G446,G447,Y449,N450,L452,E484,F490,L492,Q493,S494,P499	IEDB	[33]
	C135	10.1038/s41586-020-2852-1	7K8Z	T345,R346,S438,N439,N440,L441,P499	IEDB	[13]
	C110	10.1038/s41586-020-2852-1	7K8V	T345,R346,L441,D442,N448,Y449,N450,L452,F490,S494,Q498,P499,T500,R509	IEDB	[13]
	Fab2-7	10.1016/j.str.2021.05.014	7LSS	T345,N439,N440,S443,K444,V445,G446,G447,N450,Q498,P499,T500,Q506	IEDB	[43]
	CV07-270	10.1016/j.cell.2020.09.049	6XKP	R346,F347,S349,Y351,K444,G446,G447,N448,Y449,N450,Y451,L452,T470,E484,F490,L492,Q493,S494	IEDB	[28]
	47D1	10.1016/j.celrep.2021.109109	7MF1	R346,Y351,K444,Y449,N450,L452,T470,I472,N481,G482,V483,E484,F490,L492,S494	IEDB	[44]

	Ly-CoV1404	10.1101/2021.04.30.442182	7MMO	R346,N439,N440,S443,K444,V445,G446,G447,N450,Q498,P499,T500,N501,G502,Q506	IEDB	[45]
	REGN10987	10.1126/science.abd0827	6XDG	R346,N440,L441,K444,V445,G446,N448,Y449,Q498	IEDB	[22]
	P2B-2F6	10.1038/s41586-020-2380-z	7BWJ	R346,K444,G446,G447,N448,Y449,N450,L452,V483,E484,G485,F490,S494	IEDB	[46]
	BD-368-2'	10.1038/s41422-021-00514-9	7E8F	R346,K444,Y449,N450,L452,I472,N481,G482,V483,E484,F490,L492	IEDB	[32]
	BG1-24	10.1016/j.cell.2021.04.032	7M6I	Y351,A372,S375,T376,R408,Y449,L452,T470,I472,Y473,G482,V483,E484,G485,F486,Y489,F490,L492,Q493,S494,V503,G504,Y508	IEDB	[20]
	Fab1-57	10.1016/j.str.2021.05.014	7LS9	Y351,K444,V445,G446,G447,N448,Y449,N450,L452,T470,E484,F490,L492,Q493,S494,Q498	IEDB	[43]
	DH1043	10.1016/j.cell.2021.06.021	7LJR	Y351,G446,Y449,N450,L452,F456,T470,T478,P479,C480,N481,G482,V483,E484,G485,F486,N487,C488,Y489,F490,P491,L492,Q493,S494	IEDB	[3]
	Ly-CoV555 (Ab169)	10.1126/scitranslmed.abf1906	7KMG	Y351,Y449,L455,T470,N481,G482,V483,E484,G485,F486,C488,Y489,F490,L492,Q493,S494	IEDB	[35]
RBD-4	EY6A'	10.1016/j.cell.2021.03.055	7NXB	S366,Y369,N370,F374,F377,C379,Y380,G381,V382,S383,P384,T385,K386,N388,L390,F392,P412,D427,D428,F429,L517	IEDB	[47]
	CR3022	10.1126/science.abb7269	6W41,6YLA,6YM0	Y369,N370,S371,A372,F374,S375,T376,F377,K378,C379,Y380,G381,V382,S383,P384,T385,K386,D389,L390,F392,D427,D428,F429,T430,F515,E516,L517,H519	IEDB	[48]
	S2X259	10.1038/s41586-021-03817-4	7M7W	Y369,N370,S371,A372,F374,S375,T376,F377,K378,C379,Y380,V382,S383,P384,T385,G404,D405,R408,T500,N501,G502,V503,G504,Q506	IEDB	[49]
	S2A4	10.1016/j.cell.2020.09.037	7JVA,7JVC	Y369,N370,S371,A372,F374,S375,T376,F377,K378,C379,S383,P384,T385,R408,Q414	IEDB	[12]
	DH1047	10.1016/j.cell.2021.06.021	7LD1	Y369,N370,S371,A372,F374,S375,T376,F377,K378,C379,S383,P384,D405,R408,Q409,Q414,T415,G416,N501,V503,G504,Y505	IEDB	[3]
	COVA1-16'	10.1016/j.chom.2021.04.005	7LM8	Y369,N370,S371,F377,K378,C379,Y380,G381,V382,S383,P384,T385,R408,P412,G413,Q414,T415,G416,D427,D428,F429	IEDB	[42]
	C126	10.1016/j.celrep.2021.109604	N/A	Y369,N370,A372,F374,K378,P384	Reference Results	[50]
	H11-D4	10.1038/s41594-020-0469-6	6Z2M	Y369,N370,F374,S375,T376,F377,K378,C379,Y380,G381,V382,S383,P384,T385,K386,L390,R408,D428,T430,L517,L518	IEDB	[51]
	S304	10.1016/j.cell.2020.09.037; 10.1038/s41586-020-2349-y	7JW0,7JX3	Y369,N370,F377,K378,C379,Y380,G381,V382,S383,P384,T385,K386,N388,L390,F392,P412,G413,Q414,P426,D427,D428,F429,T430,F515,L517	IEDB	[12,41]
	COVA1-16	10.1016/j.immuni.2020.10.023	7JMW	Y369,S371,F377,K378,C379,Y380,G381,V382,S383,P384,T385,R408,P412,G413,Q414,T415,G416,D427,D428,F429	IEDB	[52]
	EY6A	10.1038/s41594-020-0480-y	6ZER,6ZFO,6ZDG,6ZDH	Y369,S375,F377,K378,C379,Y380,G381,V382,S383,P384,T385,K386,F392,P412,G413,D427,D428,F429,L517	IEDB	[53]
	CV2-75	10.1016/j.celrep.2021.109353	7M3I	F374,S375,T376,F377,K378,C379,Y380,G381,V382,S383,P384,T385,K386,R408,N437,V503,G504,Y508	IEDB	[54]
	COVOX-45	10.1016/j.cell.2021.02.032	7BEL	W353,N354,R355,K356,R357,S359,N360,N394,Y396,P426,D428,K462,P463,F464,E465,R466,I468,E516,L518,H519,A520,T523	IEDB	[2]
	S2H97	10.1038/s41586-021-03817-4	7M7W	W353,R355,R357,Y396,P426,D427,D428,F429,K462,P463,F464,R466,S514,E516,L518,H519,A520,P521	IEDB	[49]

References

1. Cerutti, G.; Guo, Y.; Zhou, T.; Gorman, J.; Lee, M.; Rapp, M.; Reddem, E.R.; Yu, J.; Bahna, F.; Bimela, J.; et al. Potent SARS-CoV-2 neutralizing antibodies directed against spike N-terminal domain target a single supersite. *Cell Host Microbe* **2021**, *29*, 819–833.e7, doi:10.1016/j.chom.2021.03.005.
2. Dejnirattisai, W.; Zhou, D.; Ginn, H.M.; Duyvesteyn, H.M.; Supasa, P.; Case, J.B.; Zhao, Y.; Walter, T.S.; Mentzer, A.J.; Liu, C.; et al. The antigenic anatomy of SARS-CoV-2 receptor binding domain. *Cell* **2021**, *184*, 2183–2200.e22, doi:10.1016/j.cell.2021.02.032.
3. Li, D.; Edwards, R.J.; Manne, K.; Martinez, D.R.; Schäfer, A.; Alam, S.M.; Wiehe, K.; Lu, X.; Parks, R.; Sutherland, L.L.; et al. In vitro and in vivo functions of SARS-CoV-2 infection-enhancing and neutralizing antibodies. *Cell l***2021**, *184*, 4203–4219.e32, doi:10.1016/j.cell.2021.06.021.
4. McCallum, M.; De Marco, A.; Lempp, F.A.; Tortorici, M.A.; Pinto, D.; Walls, A.C.; Beltramello, M.; Chen, A.; Liu, Z.; Zatta, F.; et al. N-terminal domain antigenic mapping reveals a site of vulnerability for SARS-CoV-2. *Cell* **2021**, *184*, 2332–2347.e16, doi:10.1016/j.cell.2021.03.028.
5. Voss, W.N.; Hou, Y.J.; Johnson, N.V.; Delidakis, G.; Kim, J.E.; Javanmardi, K.; Horton, A.P.; Bartzoka, F.; Paresi, C.J.; Tanno, Y.; et al. Prevalent, protective, and convergent IgG recognition of SARS-CoV-2 non-RBD spike epitopes. *Sci.* **2021**, *372*, 1108–1112, doi:10.1126/science.abg5268.
6. Chi, X.; Yan, R.; Zhang, J.; Zhang, G.; Zhang, Y.; Hao, M.; Zhang, Z.; Fan, P.; Dong, Y.; Yang, Y.; et al. A neutralizing human antibody binds to the N-terminal domain of the Spike protein of SARS-CoV-2. *Science* **2020**, *369*, 650–655, doi:10.1126/science.abc6952.
7. Wang, N.; Sun, Y.; Feng, R.; Wang, Y.; Guo, Y.; Zhang, L.; Deng, Y.-Q.; Wang, L.; Cui, Z.; Cao, L.; et al. Structure-based development of human antibody cocktails against SARS-CoV-2. *Cell Res.* **2021**, *31*, 101–103, doi:10.1038/s41422-020-00446-w.
8. Rosa, A.; Pye, V.E.; Graham, C.; Muir, L.; Seow, J.; Ng, K.W.; Cook, N.J.; Rees-Spear, C.; Parker, E.; dos Santos, M.S.; et al. SARS-CoV-2 can recruit a heme metabolite to evade antibody immunity. *Sci. Adv.* **2021**, *7*, eabg7607, doi:10.1126/sciadv.abg7607.

9. Liu, Y.; Soh, W.T.; Kishikawa, J.-I.; Hirose, M.; Nakayama, E.E.; Li, S.; Sasai, M.; Suzuki, T.; Tada, A.; Arakawa, A.; et al. An infectivity-enhancing site on the SARS-CoV-2 spike protein targeted by antibodies. *Cell* **2021**, *184*, 3452–3466.e18, doi:10.1016/j.cell.2021.05.032.
10. Kim, C.; Ryu, D.-K.; Lee, J.; Kim, Y.-I.; Seo, J.-M.; Kim, Y.-G.; Jeong, J.-H.; Kim, M.; Kim, J.-I.; Kim, P.; et al. A therapeutic neutralizing antibody targeting receptor binding domain of SARS-CoV-2 spike protein. *Nat. Commun.* **2021**, *12*, 1–10, doi:10.1038/s41467-020-20602-5.
11. Yan, R.; Wang, R.; Ju, B.; Yu, J.; Zhang, Y.; Liu, N.; Wang, J.; Zhang, Q.; Chen, P.; Zhou, B.; et al. Structural basis for bivalent binding and inhibition of SARS-CoV-2 infection by human potent neutralizing antibodies. *Cell Res.* **2021**, *31*, 517–525, doi:10.1038/s41422-021-00487-9.
12. Piccoli, L.; Park, Y.-J.; Tortorici, M.A.; Czudnochowski, N.; Walls, A.C.; Beltramello, M.; Silacci-Fregni, C.; Pinto, D.; Rosen, L.E.; Bowen, J.E.; et al. Mapping Neutralizing and Immunodominant Sites on the SARS-CoV-2 Spike Receptor-Binding Domain by Structure-Guided High-Resolution Serology. *Cell* **2020**, *183*, 1024–1042.e21, doi:10.1016/j.cell.2020.09.037.
13. Barnes, C.O.; Jette, C.A.; Abernathy, M.E.; Dam, K.-M.A.; Esswein, S.R.; Gristick, H.B.; Malyutin, A.G.; Sharaf, N.G.; Huey-Tubman, K.E.; Lee, Y.E.; et al. SARS-CoV-2 neutralizing antibody structures inform therapeutic strategies. *Nature* **2020**, *588*, 682–687, doi:10.1038/s41586-020-2852-1.
14. Barnes, C.O.; West, A.P., Jr.; Huey-Tubman, K.E.; Hoffmann, M.A.G.; Sharaf, N.G.; Hoffman, P.R.; Koranda, N.; Gristick, H.B.; Gaebler, C.; Muecksch, F.; et al. Structures of Human Antibodies Bound to SARS-CoV-2 Spike Reveal Common Epitopes and Recurrent Features of Antibodies. *Cell* **2020**, *182*, 828–842, doi:10.1016/j.cell.2020.06.025.
15. Robbiani, D.F.; Gaebler, C.; Muecksch, F.; Lorenzi, J.C.C.; Wang, Z.; Cho, A.; Agudelo, M.; Barnes, C.O.; Gazumyan, A.; Finkin, S.; et al. Convergent antibody responses to SARS-CoV-2 in convalescent individuals. *Nature* **2020**, *584*, 437–442, doi:10.1038/s41586-020-2456-9.
16. Yuan, M.; Liu, H.; Wu, N.C.; Lee, C.-C.D.; Zhu, X.; Zhao, F.; Huang, D.; Yu, W.; Hua, Y.; Tien, H.; et al. Structural basis of a shared antibody response to SARS-CoV-2. *Sci.* **2020**,*369*, 1119–1123, doi:10.1126/science.abd2321.
17. Starr, T.N.; Greaney, A.J.; Dingens, A.S.; Bloom, J.D. Complete map of SARS-CoV-2 RBD mutations that escape the monoclonal antibody LY-CoV555 and its cocktail with LY-CoV016. *Cell Rep. Med.* **2021**, *2*, 100255, doi:10.1016/j.xcrm.2021.100255.
18. Hurlburt, N.K.; Seydoux, E.; Wan, Y.-H.; Edara, V.V.; Stuart, A.B.; Feng, J.; Suthar, M.S.; McGuire, A.T.; Stamatatos, L.; Pancera, M. Structural basis for potent neutralization of SARS-CoV-2 and role of antibody affinity maturation. *Nat. Commun.* **2020**, *11*, 1–7, doi:10.1038/s41467-020-19231-9.
19. Bertoglio, F.; Fühner, V.; Ruschig, M.; Heine, P.A.; Abassi, L.; Klünemann, T.; Rand, U.; Meier, D.; Langreder, N.; Steinke, S.; et al. A SARS-CoV-2 neutralizing antibody selected from COVID-19 patients binds to the ACE2-RBD interface and is tolerant to most known RBD mutations. *Cell Rep.* **2021**, *36*, 109433, doi:10.1016/j.celrep.2021.109433.
20. Scheid, J.F.; Barnes, C.O.; Eraslan, B.; Hudak, A.; Keeffe, J.R.; Cosimi, L.A.; Brown, E.M.; Muecksch, F.; Weisblum, Y.; Zhang, S.; et al. B cell genomics behind cross-neutralization of SARS-CoV-2 variants and SARS-CoV. *Cell* **2021**, *184*, 3205–3221.e24, doi:10.1016/j.cell.2021.04.032.
21. Greaney, A.J.; Starr, T.N.; Barnes, C.O.; Weisblum, Y.; Schmidt, F.; Caskey, M.; Gaebler, C.; Cho, A.; Agudelo, M.; Finkin, S.; et al. Mapping mutations to the SARS-CoV-2 RBD that escape binding by different classes of antibodies. *Nat. Commun.* **2021**, *12*, 1–14, doi:10.1038/s41467-021-24435-8.
22. Hansen, J.; Baum, A.; Pascal, K.E.; Russo, V.; Giordano, S.; Wloga, E.; Fulton, B.O.; Yan, Y.; Koon, K.; Patel, K.; et al. Studies in humanized mice and convalescent humans yield a SARS-CoV-2 antibody cocktail. *Science* **2020**, *369*, 1010–1014, doi:10.1126/science.abd0827.
23. Du, S.; Cao, Y.; Zhu, Q.; Yu, P.; Qi, F.; Wang, G.; Du, X.; Bao, L.; Deng, W.; Zhu, H.; et al. Structurally Resolved SARS-CoV-2 Antibody Shows High Efficacy in Severely Infected Hamsters and Provides a Potent Cocktail Pairing Strategy. *Cell* **2020**, *183*, 1013–1023.e13, doi:10.1016/j.cell.2020.09.035.

24. Ge, J.; Wang, R.; Ju, B.; Zhang, Q.; Sun, J.; Chen, P.; Zhang, S.; Tian, Y.; Shan, S.; Cheng, L.; et al. Antibody neutralization of SARS-CoV-2 through ACE2 receptor mimicry. *Nat. Commun.* **2021**, *12*, 1–9, doi:10.1038/s41467-020-20501-9.
25. Yuan, M.; Huang, D.; Lee, C.-C.D.; Wu, N.C.; Jackson, A.M.; Zhu, X.; Liu, H.; Peng, L.; van Gils, M.J.; Sanders, R.W.; et al. Structural and functional ramifications of antigenic drift in recent SARS-CoV-2 variants. *Sci.* **2021**, *373*, 818–823, doi:10.1126/science.abh1139.
26. Rapp, M.; Guo, Y.; Reddem, E.R.; Yu, J.; Liu, L.; Wang, P.; Cerutti, G.; Katsamba, P.; Bimela, J.S.; Bahna, F.A.; et al. Modular basis for potent SARS-CoV-2 neutralization by a prevalent VH1-2-derived antibody class. *Cell Rep.* **2021**, *35*, 108950, doi:10.1016/j.celrep.2021.108950.
27. Cao, Y.; Su, B.; Guo, X.; Sun, W.; Deng, Y.; Bao, L.; Zhu, Q.; Zhang, X.; Zheng, Y.; Geng, C.; et al. Potent Neutralizing Antibodies against SARS-CoV-2 Identified by High-Throughput Single-Cell Sequencing of Convalescent Patients' B Cells. *Cell* **2020**, *182*, 73–84.e16, doi:10.1016/j.cell.2020.05.025.
28. Kreye, J.; Reincke, S.M.; Kornau, H.-C.; Sánchez-Sendin, E.; Corman, V.M.; Liu, H.; Yuan, M.; Wu, N.C.; Zhu, X.; Lee, C.-C.D.; et al. A Therapeutic Non-self-reactive SARS-CoV-2 Antibody Protects from Lung Pathology in a COVID-19 Hamster Model. *Cell* **2020**, *183*, 1058–1069.e19, doi:10.1016/j.cell.2020.09.049.
29. Wu, N.C.; Yuan, M.; Liu, H.; Lee, C.-C.D.; Zhu, X.; Bangaru, S.; Torres, J.L.; Caniels, T.G.; Brouwer, P.J.; van Gils, M.J.; et al. An Alternative Binding Mode of IGHV3-53 Antibodies to the SARS-CoV-2 Receptor Binding Domain. *Cell Rep.* **2020**, *33*, 108274, doi:10.1016/j.celrep.2020.108274.
30. Liu, L.; Wang, P.; Nair, M.S.; Yu, J.; Rapp, M.; Wang, Q.; Luo, Y.; Chan, J.F.; Sahi, V.; Figueroa, A.; et al. Potent neutralizing antibodies against multiple epitopes on SARS-CoV-2 spike. *Nature* **2020**, *584*, 450–456, doi:10.1038/s41586-020-2571-7.
31. Tortorici, M.A.; Beltramello, M.; Lempp, F.A.; Pinto, D.; Dang, H.V.; Rosen, L.E.; McCallum, M.; Bowen, J.; Minola, A.; Jaconi, S.; et al. Ultrapotent human antibodies protect against SARS-CoV-2 challenge via multiple mechanisms. *Sci.* **2020**, *370*, 950–957, doi:10.1126/science.abe3354.
32. Cao, Y.; Yisimayi, A.; Bai, Y.; Huang, W.; Li, X.; Zhang, Z.; Yuan, T.; An, R.; Wang, J.; Xiao, T.; et al. Humoral immune response to circulating SARS-CoV-2 variants elicited by inactivated and RBD-subunit vaccines. *Cell Res.* **2021**, *31*, 732–741, doi:10.1038/s41422-021-00514-9.
33. Dong, J.; Zost, S.J.; Greaney, A.J.; Starr, T.N.; Dingens, A.S.; Chen, E.C.; Chen, R.E.; Case, J.B.; Sutton, R.E.; Gilchuk, P.; et al. Genetic and structural basis for SARS-CoV-2 variant neutralization by a two-antibody cocktail. *Nat. Microbiol.* **2021**, *6*, 1233–1244, doi:10.1038/s41564-021-00972-2.
34. Shi, R.; Shan, C.; Duan, X.; Chen, Z.; Liu, P.; Song, J.; Song, T.; Bi, X.; Han, C.; Wu, L.; et al. A human neutralizing antibody targets the receptor-binding site of SARS-CoV-2. *Nature* **2020**, *584*, 120–124, doi:10.1038/s41586-020-2381-y.
35. Jones, B.E.; Brown-Augsburger, P.L.; Corbett, K.S.; Westendorf, K.; Davies, J.; Cujec, T.P.; Wiethoff, C.M.; Blackbourne, J.L.; Heinz, B.A.; Foster, D.; et al. The neutralizing antibody, LY-CoV555, protects against SARS-CoV-2 infection in nonhuman primates. *Sci. Transl. Med.* **2021**, *13*, doi:10.1126/scitranslmed.abf1906.
36. Koenig, P.-A.; Das, H.; Liu, H.; Kümmerer, B.M.; Gohr, F.N.; Jenster, L.-M.; Schiffelers, L.D.J.; Tesfamariam, Y.M.; Uchima, M.; Wuerth, J.D.; et al. Structure-guided multivalent nanobodies block SARS-CoV-2 infection and suppress mutational escape. *Sci.* **2021**, *371*, doi:10.1126/science.abe6230.
37. Banach, B.B.; Cerutti, G.; Fahad, A.S.; Shen, C.-H.; De Souza, M.O.; Katsamba, P.S.; Tsybovsky, Y.; Wang, P.; Nair, M.S.; Huang, Y.; et al. Paired heavy- and light-chain signatures contribute to potent SARS-CoV-2 neutralization in public antibody responses. *Cell Rep.* **2021**, *37*, 109771, doi:10.1016/j.celrep.2021.109771.
38. Clark, S.A.; Clark, L.E.; Pan, J.; Coscia, A.; McKay, L.G.; Shankar, S.; Johnson, R.I.; Brusic, V.; Choudhary, M.C.; Regan, J.; et al. SARS-CoV-2 evolution in an immunocompromised host reveals shared neutralization escape mechanisms. *Cell* **2021**, *184*, 2605–2617.e18, doi:10.1016/j.cell.2021.03.027.
39. Wu, Y.; Wang, F.; Shen, C.; Peng, W.; Li, D.; Zhao, C.; Li, Z.; Li, S.; Bi, Y.; Yang, Y.; et al. A noncompeting pair of human neutralizing antibodies block COVID-19 virus binding to its receptor ACE2. *Sci.* **2020**, *368*, 1274–1278, doi:10.1126/science.abc2241.

40. Guo, Y.; Huang, L.; Zhang, G.; Yao, Y.; Zhou, H.; Shen, S.; Shen, B.; Li, B.; Li, X.; Zhang, Q.; et al. A SARS-CoV-2 neutralizing antibody with extensive Spike binding coverage and modified for optimal therapeutic outcomes. *Nat. Commun.* **2021**, *12*, 1–11, doi:10.1038/s41467-021-22926-2.
41. Pinto, D.; Park, Y.-J.; Beltramello, M.; Walls, A.C.; Tortorici, M.A.; Bianchi, S.; Jaconi, S.; Culap, K.; Zatta, F.; De Marco, A.; et al. Cross-neutralization of SARS-CoV-2 by a human monoclonal SARS-CoV antibody. *Nat.* **2020**, *583*, 290–295, doi:10.1038/s41586-020-2349-y.
42. Liu, H.; Yuan, M.; Huang, D.; Bangaru, S.; Zhao, F.; Lee, C.-C.D.; Peng, L.; Barman, S.; Zhu, X.; Nemazee, D.; et al. A combination of cross-neutralizing antibodies synergizes to prevent SARS-CoV-2 and SARS-CoV pseudovirus infection. *Cell Host Microbe* **2021**, *29*, 806–818.e6, doi:10.1016/j.chom.2021.04.005.
43. Cerutti, G.; Rapp, M.; Guo, Y.; Bahna, F.; Bimela, J.; Reddem, E.R.; Yu, J.; Wang, P.; Liu, L.; Huang, Y.; et al. Structural basis for accommodation of emerging B.1.351 and B.1.1.7 variants by two potent SARS-CoV-2 neutralizing antibodies. *Struct.* **2021**, *29*, 655–663.e4, doi:10.1016/j.str.2021.05.014.
44. Zhou, X.; Ma, F.; Xie, J.; Yuan, M.; Li, Y.; Shaabani, N.; Zhao, F.; Huang, D.; Wu, N.C.; Lee, C.-C.D.; et al. Diverse immunoglobulin gene usage and convergent epitope targeting in neutralizing antibody responses to SARS-CoV-2. *Cell Rep.* **2021**, *35*, 109109, doi:10.1016/j.celrep.2021.109109.
45. Westendorf, K.; Wang, L.; Žentelis, S.; Foster, D.; Vaillancourt, P.; Wiggin, M.; Lovett, E.; Lee, R. van der; Hendle, J.; Pustilnik, A.; et al. LY-CoV1404 (Bebtelovimab) Potently Neutralizes SARS-CoV-2 Variants. *Biorxiv. Prepr. Serv. Biology*. **2022**, 2021.04.30.442182, doi:10.1101/2021.04.30.442182.
46. Ju, B.; Zhang, Q.; Ge, J.; Wang, R.; Sun, J.; Ge, X.; Yu, J.; Shan, S.; Zhou, B.; Song, S.; et al. Human neutralizing antibodies elicited by SARS-CoV-2 infection. *Nature* **2020**, *584*, 115–119, doi:10.1038/s41586-020-2380-z.
47. Dejnirattisai, W.; Zhou, D.; Supasa, P.; Liu, C.; Mentzer, A.J.; Ginn, H.M.; Zhao, Y.; Duyvesteyn, H.M.; Tuekprakhon, A.; Nutalai, R.; et al. Antibody evasion by the P.1 strain of SARS-CoV-2. *Cell* **2021**, *184*, 2939–2954.e9, doi:10.1016/j.cell.2021.03.055.
48. Yuan, M.; Wu, N.C.; Zhu, X.; Lee, C.-C.D.; So, R.T.Y.; Lv, H.; Mok, C.K.P.; Wilson, I.A. A highly conserved cryptic epitope in the receptor binding domains of SARS-CoV-2 and SARS-CoV. *Sci.* **2020**, *368*, 630–633, doi:10.1126/science.abb7269.
49. Tortorici, M.A.; Czudnochowski, N.; Starr, T.N.; Marzi, R.; Walls, A.C.; Zatta, F.; Bowen, J.E.; Jaconi, S.; Di Iulio, J.; Wang, Z.; et al. Broad sarbecovirus neutralization by a human monoclonal antibody. *Nat.* **2021**, *597*, 103–108, doi:10.1038/s41586-021-03817-4.
50. Chen, E.C.; Gilchuk, P.; Zost, S.J.; Suryadevara, N.; Winkler, E.S.; Cabel, C.R.; Binshtein, E.; Chen, R.E.; Sutton, R.E.; Rodriguez, J.; et al. Convergent antibody responses to the SARS-CoV-2 spike protein in convalescent and vaccinated individuals. *Cell Rep.* **2021**, *36*, 109604,, doi:10.1016/j.celrep.2021.109604.
51. Huo, J.; Le Bas, A.; Ruza, R.R.; Duyvesteyn, H.M.E.; Mikolajek, H.; Malinauskas, T.; Tan, T.K.; Rijal, P.; Dumoux, M.; Ward, P.N.; et al. Neutralizing nanobodies bind SARS-CoV-2 spike RBD and block interaction with ACE2. *Nat. Struct. Mol. Biol.* **2020**, *27*, 846–854, doi:10.1038/s41594-020-0469-6.
52. Liu, H.; Wu, N.C.; Yuan, M.; Bangaru, S.; Torres, J.L.; Caniels, T.G.; van Schooten, J.; Zhu, X.; Lee, C.-C.D.; Brouwer, P.J.; et al. Cross-Neutralization of a SARS-CoV-2 Antibody to a Functionally Conserved Site Is Mediated by Avidity. *Immun.* **2020**, *53*, 1272–1280.e5, doi:10.1016/j.immuni.2020.10.023.
53. Zhou, D.; Duyvesteyn, H.M.E.; Chen, C.-P.; Huang, C.-G.; Chen, T.-H.; Shih, S.-R.; Lin, Y.-C.; Cheng, C.-Y.; Cheng, S.-H.; Huang, Y.-C.; et al. Structural basis for the neutralization of SARS-CoV-2 by an antibody from a convalescent patient. *Nat. Struct. Mol. Biol.* **2020**, *27*, 950–958, doi:10.1038/s41594-020-0480-y.
54. Jennewein, M.F.; MacCamy, A.J.; Akins, N.R.; Feng, J.; Homad, L.J.; Hurlburt, N.K.; Seydoux, E.; Wan, Y.-H.; Stuart, A.B.; Edara, V.V.; et al. Isolation and characterization of cross-neutralizing coronavirus antibodies from COVID-19+ subjects. *Cell Rep.* **2021**, *36*, 109353, doi:10.1016/j.celrep.2021.109353
